# Modulating the microenvironment during FVIII uptake influences the nature of FVIII-peptides presented by antigen-presenting cells

**DOI:** 10.3389/fimmu.2022.975680

**Published:** 2022-10-21

**Authors:** Christian Lubich, Katharina Nora Steinitz, Brigitte Hoelbl, Thomas Prenninger, Pauline Maria van Helden, Markus Weiller, Birgit Maria Reipert

**Affiliations:** ^1^ R & D, Baxalta Innovations GmbH, a member of the Takeda group of companies, Vienne, Austria; ^2^ Institute Krems Bioanalytics, University of Applied Sciences Krems, Krems, Austria

**Keywords:** FVIII presentation by antigen-presenting cells, FVIII-specific CD4+ T-cell hybridoma library, HLA-DRB1*1501 humanized hemophilic mice, Hemophilia A, neutralizing anti-drug antibodies, FVIII inhibitors, microenvironment during FVIII uptake by antigen-presenting cells

## Abstract

**Background and aims:**

Hemophilia A is a severe bleeding disorder caused by the deficiency of functionally active coagulation factor VIII (FVIII). The induction of neutralizing anti-drug antibodies is a major complication in the treatment of hemophilia A patients with FVIII replacement therapies. Why some patients develop neutralizing antibodies (FVIII inhibitors) while others do not is not well understood. Previous studies indicated that the induction of FVIII inhibitors requires cognate interactions between FVIII-specific B cells and FVIII-specific CD4+ T cells in germinal center reactions. In this study, we investigated the FVIII peptide repertoire presented by antigen-presenting cells (APCs) under different microenvironment conditions that are expected to alter the uptake of FVIII by APCs. The aim of this study was to better understand the association between different microenvironment conditions during FVIII uptake and the FVIII peptide patterns presented by APCs.

**Methods:**

We used a FVIII-specific CD4+ T cell hybridoma library derived from humanized HLA-DRB1*1501 (human MHC class II) hemophilic mice that were treated with human FVIII. APCs obtained from the same mouse strain were preincubated with FVIII under different conditions which are expected to alter the uptake of FVIII by APCs. Subsequently, these preincubated APCs were used to stimulate the FVIII-specific CD4+ T cell hybridoma library. Stimulation of peptide-specific CD4+ T-cell hybridoma clones was assessed by analyzing the IL-2 release into cell culture supernatants.

**Results:**

The results of this study indicate that the specific microenvironment conditions during FVIII uptake by APCs determine the peptide specificities of subsequently activated FVIII-specific CD4+ T cell hybridoma clones. Incubation of APCs with FVIII complexed with von Willebrand Factor, FVIII activated by thrombin or FVIII combined with a blockade of receptors on APCs previously associated with FVIII uptake and clearance, resulted in distinct peptide repertoires of subsequently activated hybridoma clones.

**Conclusion:**

Based on our data we conclude that the specific microenvironment during FVIII uptake by APCs determines the FVIII peptide repertoire presented on MHC class II expressed by APCs and the peptide specificity of subsequently activated FVIII-specific CD4+ T cell hybridoma clones.

## Introduction

Hemophilia A is a congenital bleeding disorder characterized by the lack or insufficient amount of functional factor VIII (FVIII) in the circulation which results in prolonged bleeding episodes due to ineffective blood coagulation upon injury (1). Most patients with severe hemophilia A receive FVIII replacement therapies (2). About 30% of these patients develop neutralizing anti-drug antibodies against FVIII (3), so called FVIII inhibitors, which remains the major challenge in FVIII replacement therapies (4). Although research has greatly contributed to our understanding of the risk factors for the formation of FVIII inhibitors (5), the reason why some patients develop inhibitors and others do not is not well understood. Several studies in hemophilia A patients and in murine models of hemophilia A suggested that FVIII-specific CD4+ T cells are crucial for the induction of neutralizing antibody responses against FVIII (6–9). CD4+ T cells express T-cell receptors that recognize antigen-derived peptides (CD4+ T cell epitopes) presented by MHC class II molecules which are expressed on specialized antigen-presenting cells (APCs) (10). Three major mechanisms contribute to the uptake of proteins such as FVIII by APCs, namely non-selective macropinocytosis, receptor-mediated endocytosis and phagocytosis (11). After their uptake, proteins are sorted into different compartments of the endosomal pathway, where they are processed into peptides and subsequently loaded onto MHC class II molecules. Peptide/MHC class II complexes are redistributed to the limiting membrane of the vesicle and moved into tubulovesicular structures that fuse directly with the plasma membrane (12). This process facilitates the presentation of peptide-MHC class II complexes on the surface of APCs. CD4+ T cells bind to these complexes *via* their specific T cell receptor (TCR), recognizing specific peptides presented by MHC class II, and by the CD4 receptor on T cells (11).

The diversity of the peptide repertoire presented by APCs depends both on the enzymes encountered by protein antigens during processing in endosomal compartments and on the receptivity of the MHC class II molecules (13). Both these parameters depend on the type of endosomal compartment(s) to which the protein antigens are sorted for processing and loading onto MHC class II molecules.

Several authors described a modulation of anti-FVIII immune responses as a result of preventing FVIII endocytosis by APCs. Dasgupta and colleagues presented data indicating that von Willebrand Factor (VWF), the plasma chaperon of FVIII, prevents FVIII uptake by human monocyte-derived dendritic cells which resulted in a reduced activation of a FVIII-specific CD4+ T cell clone *in vitro* (14). In addition, the authors demonstrated that the inhibition of FVIII uptake by APCs depends on the actual binding of FVIII to VWF. A mutated VWF protein which lacks the binding site for FVIII did not influence the endocytosis of FVIII (14). More recently, Sorvillo and co-workers showed that VWF, complexed with FVIII, binds to human dendritic cells which results in the modulation of FVIII-derived peptide patterns presented on MHC-class II at the surface of these cells (15). These data suggest that VWF does not completely block FVIII uptake by APCs but rather modulates FVIII processing and presentation. In another study, the same authors indicated that blocking specific residues in the C1 domain of FVIII, that are involved in the interaction of FVIII with phospholipid membrane surfaces, not only inhibits FVIII endocytosis *in vitro* but also reduces the immune response to FVIII in hemophilic mouse models *in vivo* (16). These data suggest a critical role of the C1 domain in the endocytosis of FVIII by APCs and the subsequent processing and presentation of FVIII peptides on MHC class II.

Depending on the route of endocytosis, FVIII can potentially be directed to different endosomal compartments, where multiple factors such as protein unfolding and proteolysis contribute to the process of epitope selection by MHC class II molecules (17, 18). Therefore, it is important to understand which FVIII peptides are presented by MHC class II complexes on APCs under conditions of FVIII replacement therapies. In the circulation, endogenous FVIII binds to several proteins during the three major stages of its life cycle: transport of FVIII from the site of secretion to the bleeding site, participation of FVIII in the coagulation cascade, and clearance of FVIII from the circulation (19). Factor VIII circulates as a mixture of heterodimers formed as a result of proteolysis at the B-A3 junction and additional cleavages within the B domain (17). After FVIII is released into the circulation it immediately forms a non-covalent complex with VWF, predominantly *via* the a3 acidic peptide and the A3 and C2 domains (18). VWF not only protects FVIII from further proteolytic cleavage, but also directs FVIII to the subendothelium and to activated platelets after vascular injury (20). At the site of bleeding, thrombin, proteolytically activated from circulating prothrombin and released from activated platelets, activates FVIII by cleavages at Arg372, Arg740 and Arg1689 within the acidic regions (a1, a2, and a3) neighboring the A domains (21). Thrombin-activated FVIII (FVIIIa) transiently circulates as a heterotrimer consisting of A1, A2, and A3-C1-C2 after the B domain is released (22). Upon FVIII activation, VWF is released and FVIIIa forms the so called tenase complex together with FIXa, FX and phospholipids (17, 23). After activation of FX to FXa, the tenase complex dissembles and FVIIIa gets deactivated by spontaneous dissociation of its subunits or *via* further proteolytic cleavages by activated protein C (24). Several catabolic receptors on scavenger cells have been associated with the clearance of native, activated or inactivated FVIII: the asialoglycoprotein receptors (ASGPR), the macrophage mannose receptors (MMR), receptors of the LDL receptor (LDLR) family and heparan sulfate proteoglycans (HSPG) (16, 25–31).

In this study, we investigated the recognition of FVIII peptides by FVIII-specific CD4+ T cell hybridoma clones under different experimental microenvironment conditions that are expected to alter the uptake of FVIII by APCs. The aim of this study was to better understand the association between different microenvironment conditions during FVIII uptake and the FVIII peptide patterns presented by APCs. In order to address this question, we compared FVIII with FVIII complexed with VWF, FVIII activated by thrombin, and FVIII accompanied by a concurrent blockade of different receptors on APCs previously associated with FVIII uptake and clearance. Moreover, we used a recently generated, comprehensive FVIII-specific CD4+ T cell hybridoma library that was derived from the E17 humanized HLA-DRB1*1501 transgenic hemophilic mouse model (32). The hemophilic mice of this model express the human MHC-class II protein HLA-DRB1*1501 on the background of a complete knockout of all murine MHC class II proteins (33). We used this library as a tool to monitor FVIII processing and presentation by APCs, by comparing the FVIII peptide specificities of the subsequently activated FVIII-specific CD4+ T cell hybridoma clones.

## Methods

### Humanized E17 HLA-DRB1*1501 mice

Humanized E17 HLA-DRB1*1501 mice, as described by Reipert et al. (33), are characterized by a chimeric expression of the human MHC class II HLA-DRB1*1501 with the human sequences for the binding of peptides and the murine sequence for the binding of murine CD4 in combination with a knockout of all mouse MHC class II proteins and a hemophilic E17 FVIII knockout as described by Bi et al. (34).

### Treatment of mice with recombinant human FVIII

All studies were performed in accordance with Austrian federal law (Act BGBl. I No. 114/2012) regulating animal experimentation and approved by the Institutional Animal Care and Use Committee of Baxalta Innovations GmbH, a member of the Takeda group of companies, Vienna, Austria. Mice were male and 8 to 12 weeks old at the beginning of the experiments. The mice received up to 9 weekly intravenous or subcutaneous doses of 1 µg recombinant human full-length FVIII (Baxalta Innovations GmbH, a member of the Takeda group of companies, Vienna, Austria).

### Generation of FVIII-specific T cell hybridoma clones

T cell hybridoma clones were generated as described by Steinitz et al. (32). Mice were treated as outlined above and spleens were obtained three days after the last dose of human FVIII. The splenocytes were prepared as described previously (35) and re-stimulated either with 20 µg/mL human recombinant FVIII or with 1 µg/peptide/mL of a human FVIII peptide pool (Charité Berlin, Germany) for 3 days at 37°C and 5% CO2. The peptide pool consisted of 774 15mer FVIII peptides with an offset of 3 amino acids. The re-stimulated splenocytes were fused to BW5147 cells (BW5147.G.1.4; ATCC #TIB 48) using polyethylene glycol fusion (36), plated into 96 well plates and cultured for 2 days at 37°C and 5% CO2 prior to a two weeks selection with hypoxanthine-aminopterin-thymidine (HAT media supplement, Sigma Aldrich, Missouri USA). Cells that grew under selection media were picked, screened for FVIII specificity and subcloned *via* limiting dilution. Core peptides were predicted using NetMHCIIpan-3.1 (37).

### Screening for FVIII-specific hybridoma clones

The specificity of hybridoma clones for FVIII was assessed by coculturing a total of 1 x 10^5^ hybridoma cells with whole splenocytes, used as APCs, derived from naïve mice for 24 hours at 37°C and 5% CO2, in the presence of either 10 µg/mL human FVIII or human FVIII peptide pools (Charité Berlin, Germany), respectively.

IL-2 release into cell culture supernatants was used as the readout for the activation of hybridoma clones by APCs preincubated with FVIII or FVIII peptide pools, respectively. IL-2 release was analyzed using either an IL-2 ELISA (Biolegend, California USA) or an IL-2 Bio-Plex assay (Bio-Rad Laboratories, California USA). Hybridoma clones that responded with an at least 5-fold increase in IL-2 release (ratio between FVIII-stimulated cultures and medium controls) were considered to be FVIII-specific and subsequently subcloned *via* limiting dilution.

### Assessment of FVIII peptide specificity of FVIII-specific hybridoma clones

The FVIII peptide specificity of FVIII-specific hybridoma clones was identified using cross matrix schemes as previously described (38). Here, two identical peptide membranes, each spanning the whole FVIII molecule, were synthesized and one cut in rows and the other cut in columns. The peptides from each row and each column were dissolved in DMSO (Sigma Aldrich, Missouri USA) into individual pools containing up to 33 FVIII peptides (1 µg/mL for each peptide). Each T-cell hybridoma was tested against all peptide pools that were processed from the vertical and horizontal strips. The crossing point of positive hits for the vertical and the horizontal pools revealed the peptide that was recognized by a particular T-cell hybridoma clone (38).

### Preparation of different APC populations from mouse spleens

If not otherwise indicated, whole splenocytes were used as APCs in most experiments.

In some experiments whole splenocytes (APCs) were compared with different splenic APC populations. Different APC populations were prepared as follows:

#### Whole splenocytes

Spleens were finely minced and passed through a 70-μM nylon cell strainer (Becton Dickinson, Franklin Lakes, NJ). Spleen cells were collected in RPMI 1640 (Life Technologies, Paisley, Scotland) supplemented with 10% preselected fetal calf serum (FCS), 2 mM l-glutamine (both from Hyclone, Logan, UT), 100 U/mL penicillin, 100 mg/mL streptomycin (both from Life Technologies), and 5 × 10–5 M β-mercaptoethanol (Sigma-Aldrich). Single-cell suspensions were cleared of erythrocytes by hemolysis using a hypotonic buffer (pH 7.2) composed of 0.15 M ammonium chloride, 10 mM potassium bicarbonate (both from Merck, Darmstadt, Germany), and 0.1 mM ethylene-diaminetetraacetic acid (Life Technologies).

To evaluate the composition of APCs, splenocytes were stained for CD3e PerCP-eFluor 710 (clone 17A2, Thermo Fisher Scientific), CD19 FITC (clone 1D3, BD Biosciences), CD11c APC (HL3, BD Biosciences), CD11b PE-Cy7 (clone M1/70, Thermo Fisher Scientific) and HLA-DR BV605 (clone G46-6, BD Biosciences) and analyzed using a BD FACS Aria III (BD Biosciences) and FlowJo software (Version 10.8.1; BD Biosciences). Nonspecific binding through Fc gamma receptors was blocked by a mixture of anti-CD16 and anti-CD32 antibodies (Fc Block; BD Biosciences).

#### Splenic CD19+ B cells

Splenocytes were prepared as described before. Afterwards, cells were stained for CD3e PerCP-eFluor 710 (clone 17A2, Thermo Fisher Scientific), CD19 FITC (clone 1D3, BD Biosciences), CD11c APC (HL3, BD Biosciences), CD11b PE-Cy7 (clone M1/70, Thermo Fisher Scientific) and HLA-DR BV605 (clone G46-6, BD Biosciences) and subsequently sorted using a BD FACS Aria III (BD Biosciences). Post sort analysis using FlowJo software (Version 10.8.1; BD Biosciences) showed a purity of >98% for splenic CD19+ B cells.

#### Splenic CD11b+ monocyte/macrophages

Splenocytes were prepared as described before. Monocytes/macrophages were enriched by a pre-isolation step using NKp46, CD3e and CD19 MACS beads negative selection (all Miltenyi Biotech, Germany). Subsequently, cells were stained for CD3e PerCP-eFluor 710 (clone 17A2, Thermo Fisher Scientific), CD19 FITC (clone 1D3, BD Biosciences), CD11c APC (HL3, BD Biosciences), CD11b PE-Cy7 (clone M1/70, Thermo Fisher Scientific) and HLA-DR BV605 (clone G46-6, BD Biosciences) and sorted using a BD FACS Aria III (BD Biosciences). Nonspecific binding through Fc gamma receptors was blocked by a mixture of anti-CD16 and anti-CD32 antibodies (Fc Block; BD Biosciences). Post sort analysis using FlowJo software (Version 10.8.1; BD Biosciences) showed an average purity of 94.4% for splenic CD11b+ monocytes/macrophages.

#### Splenic CD11c high MHC class II+ dendritic cells

Splenocytes were prepared as described before. Dendritic cells were enriched by a pre-isolation step using NKp46, CD3e and CD19 MACS beads negative selection (all Miltenyi Biotech, Germany). Subsequently, cells were stained for CD3e PerCP-eFluor 710 (clone 17A2, Thermo Fisher Scientific), CD19 FITC (clone 1D3, BD Biosciences), CD11c APC (HL3, BD Biosciences), CD11b PE-Cy7 (clone M1/70, Thermo Fisher Scientific) and HLA-DR BV605 (clone G46-6, BD Biosciences) and sorted using a BD FACS Aria III (BD Biosciences). Nonspecific binding through Fc gamma receptors was blocked by a mixture of anti-CD16 and anti-CD32 antibodies (Fc Block; BD Biosciences). Post sort analysis using FlowJo software (Version 10.8.1; BD Biosciences) showed an average purity of 94.8% for splenic CD11c high MHC class II+ dendritic cells.

### Generation of thrombin-activated FVIII

The generation of human thrombin-activated FVIII was performed as described by Pfistershammer et al. (39). In short, 60 units of human thrombin (Sigma Aldrich, Missouri USA) were added to 3000 units of human recombinant FVIII in 15 mL of RPMI 1640 (Invitrogen, California USA) and incubated at 37°C for 30 minutes. Subsequently the thrombin inhibitor Pefabloc TH (Pentapharm, Switzerland) was added at a final concentration of 10 µM.

### Monitoring alterations in FVIII antigen processing using the FVIII specific CD4+ T cell hybridoma library

The FVIII peptide repertoire presented by APCs was monitored by analyzing the peptide specificities of subsequently activated FVIII specific CD4+ T cell clones. In detail, APCs from naïve HLA-DRB1*1501 mice were incubated with human recombinant FVIII, thrombin activated FVIII (FVIIIa) or FVIII in complex with human purified plasma-derived VWF. Equal units of human FVIII and human VWF were co-incubated in serum-free medium at room temperature for one hour to facilitate complex building. As a control, FVIII was co-incubated with Human Serum Albumin (HAS, Sigma Aldrich, Missouri USA) using the same protein concentrations as used for VWF. Preincubation with APCs was done at 37°C and 5% CO2 for 3 hours to allow protein uptake. Subsequently, APCs were washed twice to remove remaining protein in the medium. Afterwards, APCs were added to FVIII-specific CD4+ T cell clones and incubated at 37°C 5% CO2. After 48 hours the supernatants were collected and analyzed for IL-2 release, a marker for the activation of CD4+ T cell clones, using a Bio-Plex assay (Bio-Rad Laboratories, California USA).

In some experiments, FVIII receptor blocking agents were added to APC cultures 30 min prior to the addition of FVIII and the subsequent 3 hours culture for FVIII uptake. The following blocking agents were used as indicated: 5mM Galactose, 100µg/mL Heparin, 2mg/mL Mannan (all three: Sigma Aldrich, Missouri USA), 10µg/mL blocking monoclonal antibody against Mannose Receptor (Abcam, UK), 14.4 µg/mL recombinant mouse LRPAP1 (Sino Biological, China), 1.25mM and 5mM EDTA (Amresco, Ohio USA).

All experiments were done in triplicates.

### Statistical analysis

Statistical analysis for significance between different groups of measured mean IL-2 release was assessed by a two-way ANOVA with Sidaks’s multiple comparison test using Graphpad Prism 9 (GraphPad Software, Inc., California USA). P < 0.05 was considered statistically significant.

## Results

### Expansion of the FVIII-specific CD4+ T cell hybridoma library utilizing humanized E17 HLA-DRB1*1501 mice

**Figure 1:**
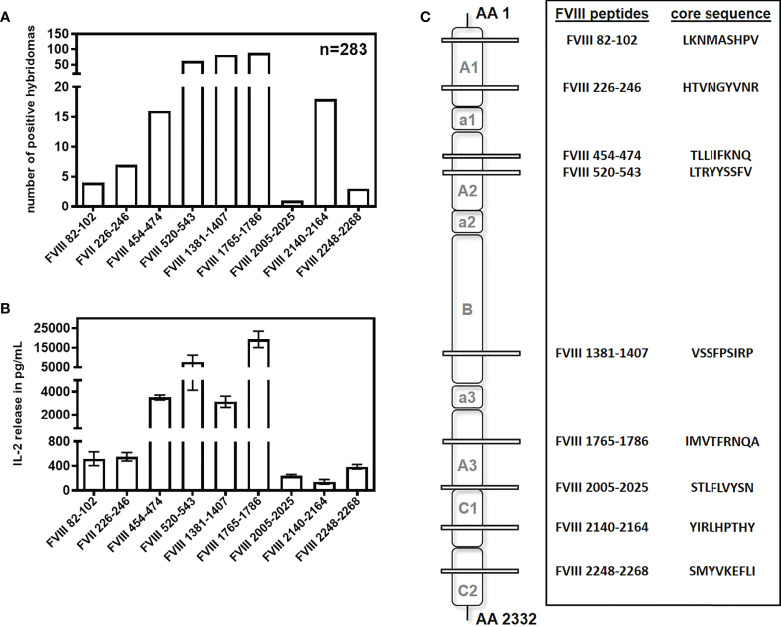
Repertoire of CD4+ T cell epitopes after intravenous and subcutaneous application of FVIII in humanized E17 HLA-DRB1*1501 mice. E17 HLADRB1*1501 mice (E17 human MHC class II) received up to 9 weekly intravenous or subcutaneous doses of 1 µg recombinant human full-length FVIII. Spleen cells were obtained 3 days after the last immunization, re-stimulated with either human FVIII protein or a human FVIII peptide pool, and subsequently fused with cells of a BW thymoma cell line as described (32). CD4+ T cell hybridoma clones were characterized for their peptide specificity using a 15-mer peptide library (12 amino acids overlap) covering the sequence of full-length human FVIII. **(A)** Number and peptide specificity of CD4+ T cell hybridoma clones obtained from a total of 24 fusion campaigns. **(B)** Representative examples (n=3) for the IL-2 release of FVIII-specific CD4+ T cell hybridoma clones specific for different FVIII peptide regions during incubation with 10U FVIII. Presented is the mean value ± SD. **(C)** Core sequences, peptide specificities and associated FVIII domains of the CD4+ T cell hybridoma clones described in **(A)**.

Previously, we reported the major FVIII peptide regions containing T-cell epitopes involved in the immune response of humanized E17 HLA-DRB1*1501 mice against human FVIII (32). In an attempt to expand the hybridoma library and increase the repertoire of unique FVIII peptide-specific CD4+ T-cell hybridoma clones, we slightly modified the procedure for the generation of FVIII-specific T cell hybridoma clones. Instead of using whole FVIII protein in the first *in vitro* restimulation of splenocytes obtained from mice treated with up to 9 doses of human FVIII, we used a whole human FVIII peptide pool consisting of 15-mer peptides shifted by 3 amino acids. This way, we generated 99 novel FVIII-specific CD4+ T cell hybridoma clones. Moreover, we identified a new CD4+ T-cell epitope in the C2 domain of FVIII (FVIII 2248-2268) which complemented the 8 epitopes that we had already reported in Steinitz et al. (32). The new FVIII-specific CD4+ T cell hybridoma clones expanded our FVIII-specific hybridomas library obtained from humanized E17 HLA-DRB1*1501 mice to a total of 283 CD4+ T cell hybridoma clones recognizing 9 different epitope regions (**Figure 1**). We used this library for all subsequent studies related to potential alterations of FVIII-peptide presentations under different microenvironment conditions that are expected to alter the uptake of FVIII by APCs.

**Figure 2:**
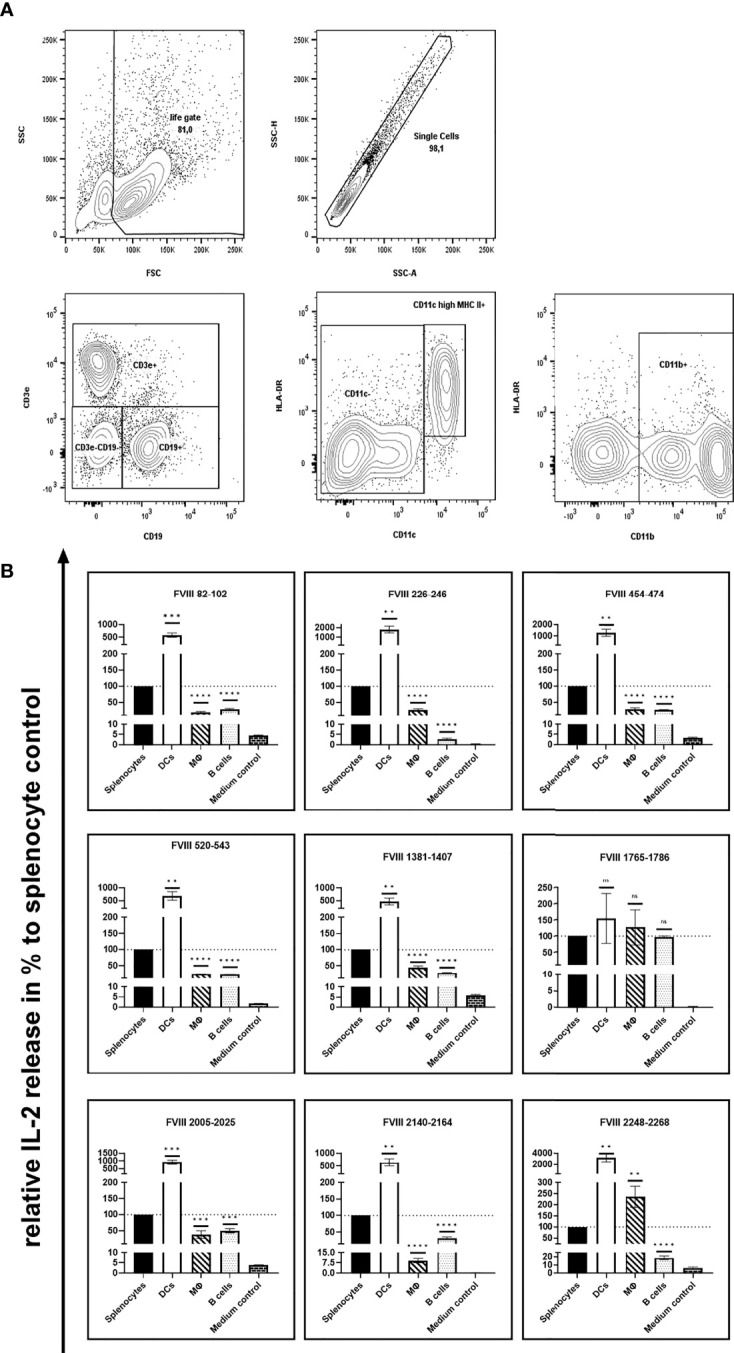
Capacity of different APC populations to present FVIII peptides and subsequently activate FVIII-specific CD4+ T cell hybridoma clones. FVIII specific CD4+ T cell hybridoma clones were cocultured with total splenocytes, purified splenic CD11c high MHC class II+ dendritic cells, purified splenic CD11b+ monocytes/macrophages or purified splenic CD19+ B cells preincubated with human FVIII or Medium control, as indicated. After 24 hours coculture of preincubated APCs with CD4+ T cell hybridoma clones, culture supernatants were collected and analyzed for IL-2 release as an indicator for the activation of the CD4+ T cell hybridoma clones. **(A)** Gating strategy for the purification of splenic CD11c high MHC class II+ dendritic cells, splenic CD11b+ monocytes/macrophages and splenic CD19+ B cells. After exclusion of dead cells and doublets and subsequent selection of lymphocytes based on physical parameters, splenic B lymphocytes were identified by expression of CD19. Splenic dendritic cells and monocytes/macrophages were gated on CD3e/CD19 double negative cells and further discriminated by expression of CD11c high/HLA-DR or CD11b, respectively. **(B)**The IL-2 release is shown as relative IL-2 release in %, compared to the IL-2 release of the respective splenocyte control group (100%). Depicted is the mean value ± SEM. Statistical analysis for significance between different APCs and the splenocyte control group of measured mean IL-2 release was assessed by an unpaired t-test using Graphpad Prism 9 (GraphPad Software, Inc., California USA). P < 0.05 was considered statistically significant. ns means not significant; *p<0.05; **p<0.01; ***p<0.001; ****p<0.0001. Detailed data are provided in **Table 1**. All experiments were done in triplicates. For comparison, absolute values for the related IL-2 release induced by splenocyte control APCs are presented in **Figure 1B**.

As the route of FVIII uptake and the subsequent processing of FVIII might differ between distinct populations of APCs, we were interested in the capability of different splenic APC populations to efficiently present the identified FVIII peptides. To address this question, we isolated CD11c high MHC class II+ dendritic cells, CD11b+ monocytes/macrophages and CD19+ B cells using splenocytes isolated from naïve humanized E17 HLA-DRB1*1501 mice and analyzed the stimulation of peptide-specific CD4+ T cell hybridoma clones by the different APC populations in the presence of 10µg/mL human FVIII. Results depicted in **Figure 2** and **Table 1** show differences in the capacity of the different APC populations to activate the respective FVIII-specific CD4+ T cell hybridoma clones. CD11c high MHC class II+ dendritic cells outperformed the other APC populations investigated in their capacity to activate the FVIII-specific CD4+ T cell hybridoma clones for most clones, except for clones specific for peptide FVIII 1765-1786 in the A3 domain. All APC populations investigated were able to activate CD4+ T cell hybridoma clones specific for the respective epitopes, as indicated by the induction of an IL-2 release which was at least 5-10-fold higher than in the respective medium control (**Figure 2** and **Table 1**).

**Table 1:** Capability of different APC populations to present FVIII peptides and subsequently activate FVIII-specific CD4+ T cell hybridoma clones.

	DCs(CD11c high MHCII+ cells)	Monocytes/Macrophages(CD11b+)	B cells(CD19+ cells)	Medium control
	Relative IL-2 release in %, compared to total splenocytes ( ± SEM)			
FVIII 82-102	580.50 ( ± 47.54)	19.12 ( ± 1.19)	29.19 ( ± 1.27)	4.63 ( ± 0.10)
FVII 226-246	1793.83 ( ± 206.97)	26.34 ( ± 2.32)	2.76 ( ± 0.29)	0.43 ( ± 0.08)
FVIII 454-474	1275.58 ( ± 183.85)	28.36 ( ± 2.21)	25.61 ( ± 0.80)	3.30 ( ± 0.17)
FVIII 520-543	685.93 ( ± 97.01)	23.40 ( ± 0.49)	21.78 ( ± 0.35)	1.70 ( ± 0.08)
FVIII 1381-1407	468.47 ( ± 79.57)	44.54 ( ± 2.85)	28.14 ( ± 0.57)	5.93 ( ± 0.22)
FVIII 1765-1786	154.38 ( ± 44.43)	126.77 ( ± 31.40)	96.42 ( ± 2.23)	0.31 ( ± 0.04)
FVIII 2005-2025	937.91 ( ± 73.10)	38.16 ( ± 7.01)	49.43 ( ± 3.93)	3.82 ( ± 0.15)
FVIII 2140-2164	642.34 ( ± 80.77)	9.21 ( ± 0.96)	31.36 ( ± 2.10)	0.34 ( ± 0.04)
FVIII 2248-2268	3191.72 ( ± 448.05)	235.72 ( ± 27.37)	18.58 ( ± 1.23)	5.74 ( ± 0.96)

Presented is the relative IL-2 release in percent, compared to the IL-2 release in the respective total splenocyte control group (100%). All experiments were done in triplicates. Absolut values for the IL-2 release induced by the total splenocyte control group are presented in **Figure 1B**.

### VWF has distinct effects on the presentation of some but not all FVIII peptides investigated

**Figure 3:**
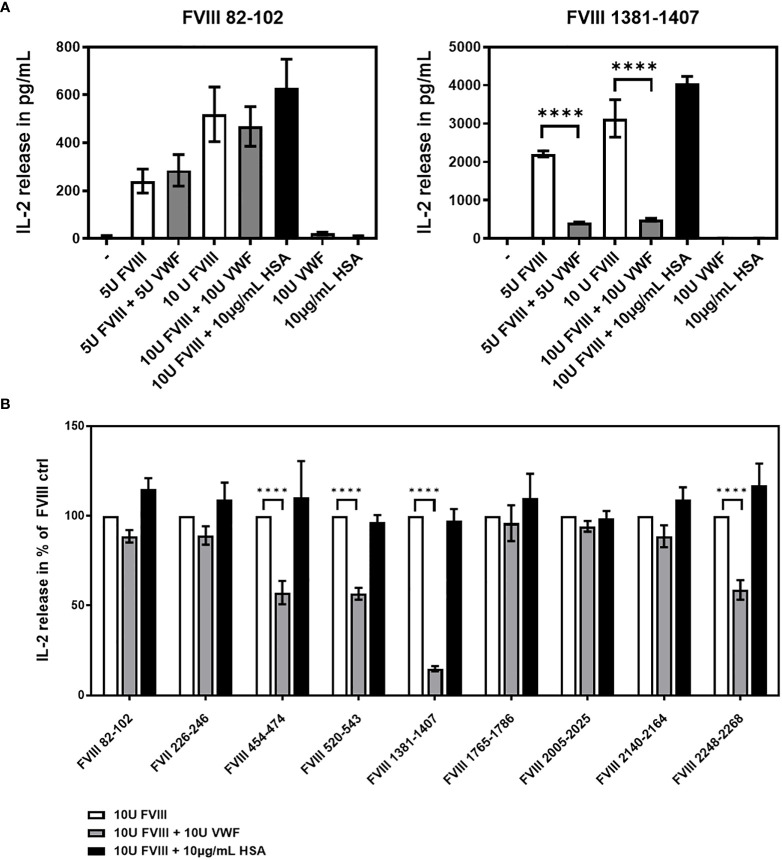
VWF present during preincubation of APCs modulates presentation of FVIII-derived peptides and subsequent stimulation of peptide-specific CD4+ T cells hybridoma clones. FVIII specific CD4+ T cell hybridoma clones were cocultured with APCs preincubated with either human FVIII only or a complex of human FVIII with human VWF. As a control, FVIII was mixed with Human Serum Albumin in the same protein concentration as used for VWF. After 48 hours culture of preincubated APCs with CD4+ T cell hybridoma clones, culture supernatants were collected and analyzed for IL-2 release as an indicator for the activation of the CD4+ T cell hybridoma clones. **(A)** Two representative examples for the influence of VWF present during the preincubation of APCs on the subsequent activation of FVIII-specific CD4+ T cell hybridoma clones specific for different FVIII peptide regions. Presented is the mean value ± SD **(B)** Overview of the influence of VWF present during the preincubation of APCs on the subsequent activation of FVIII-specific CD4+ T cell hybridoma clones specific for the different FVIII peptide regions tested. Depicted is the mean value ± SEM. Results were derived from 6 independent experiments, each done in triplicates (n=18). Statistical analysis for significance between different groups of measured mean IL-2 release was assessed by a two-way ANOVA with Sidaks’s multiple comparison test using Graphpad Prism 9 (GraphPad Software, Inc., California USA). P < 0.05 was considered statistically significant. *p<0.05; **p<0.01; ***p<0.001; ****p<0.0001. Detailed data including statistical analysis are depicted in **Table 2**.

Next, we asked if human VWF modulates the presentation of FVIII-derived peptides and the subsequent stimulation of peptide-specific CD4+ T cell hybridoma clones derived from the FVIII-specific CD4+ T cell hybridoma library. To address this question, APCs obtained from naïve humanized E17 HLA-DRB1*1501 mice were incubated with either human FVIII or a complex of human FVIII and human VWF and subsequently co-cultured with the FVIII-specific CD4+ T cell hybridoma library. The composition of APCs as evaluated by flow cytometry, indicated that the APCs contained approximately 30% splenic CD19+ B cells, 4.5% splenic CD11c high MHC class II+ dendritic cells and 7.5% splenic CD11b+ monocytes/macrophages. IL-2 released into culture supernatants of each hybridoma clone co-culture with APCs was analyzed. As a protein control, we included Human Serum Albumin (HSA) at the highest protein concentration used for co-cultures with human VWF. The results of two representative examples for the potential influence of VWF on the IL-2 release by clones of the FVIII-specific CD4+ T cell hybridoma library are shown in **Figure 3A**. While the IL-2 release by the FVIII-specific CD4+ T cell hybridoma clone specific for the FVIII peptide region 82-102 within the A1 domain of FVIII was not affected by VWF, the data indicate an almost complete reduction in IL-2 release by the FVIII-specific CD4+ T cell hybridoma clone specific for the FVIII peptide region 1381-1407 within the B domain. In the experiments presented in **Figure 3A**, we used either 5U/mL or 10 U/mL of FVIII in the presence or absence of 5U/mL or 10 U/mL of VWF, respectively. We also tested a clinically more relevant dose of 1U/mL FVIII with or without 1U/mL VWF. We observed similar trends as presented in **Figure 3A** but the overall stimulation of peptide-specific CD4+ T cells was weak in comparison to the stimulation observed when using 5 or 10 U/mL of FVIII and VWF. Overall, the presence of VWF during incubation of APCs with FVIII significantly (p < 0,0001) reduced the subsequent activation of CD4+ T-cell hybridoma clones specific for 4 of the 9 identified CD4+ T-cell epitopes (**Figure 3B** and **Table 2**). The reduction was about 85% in the clones specific for the B domain peptide (FVIII 1381-1407) and about 40-50% in two clones specific for the A2 domain (FVIII 454-475 and FVIII 520-544) and one clone specific for the C2 domain (FVIII 2248-2268). However, the activation of CD4+ T-cell hybridoma clones specific for the remaining 5 of the 9 identified CD4+ T-cell epitopes was not modified by the presence of VWF in the preincubation of APCs (**Figure 3B** and **Table 2**).

**Table 2:** Influence of VWF present during preincubation of APCs with FVIII on the peptide specificity of subsequently activated FVIII-specific CD4+ T cell hybridoma clones.

FVIII T-cell epitope	10U FVIII + 10U VWF (mean ± SEM)	p value	Significance
FVIII 82-102	88.53 ( ± 3.46)	0.285	ns
FVII 226-246	89.04 ( ± 5.12)	0.3432	ns
FVIII 454-474	57.25 ( ± 6.46)	< 0.0001	****
FVIII 520-543	56.54 ( ± 3.32)	< 0.0001	****
FVIII 1381-1407	14.82 ( ± 1.44)	< 0.0001	****
FVIII 1765-1786	95.88 ( ± 9.95)	0.9955	ns
FVIII 2005-2025	94.16 ( ± 2.98)	0.9515	ns
FVIII 2140-2164	88.59 ( ± 6.13)	0.2914	ns
FVIII 2248-2268	58.72 ( ± 5.48)	< 0.0001	****

Presented is the relative IL-2 release in percent, compared to the IL-2 release in the respective “FVIII only” control group (100%). Statistical analysis for significance of the differences in IL-2 release between each of the “FVIII only” control groups and the related “FVIII+VWF” group were assessed by a two-way ANOVA with Sidaks’s multiple comparison test using Graphpad Prism 9. P < 0.05 was considered statistically significant. *p<0.05; **p<0.01; ***p<0.001; ****p<0.0001.

### Thrombin activation of FVIII alters the FVIII peptide pattern presented by APCs

**Figure 4:**
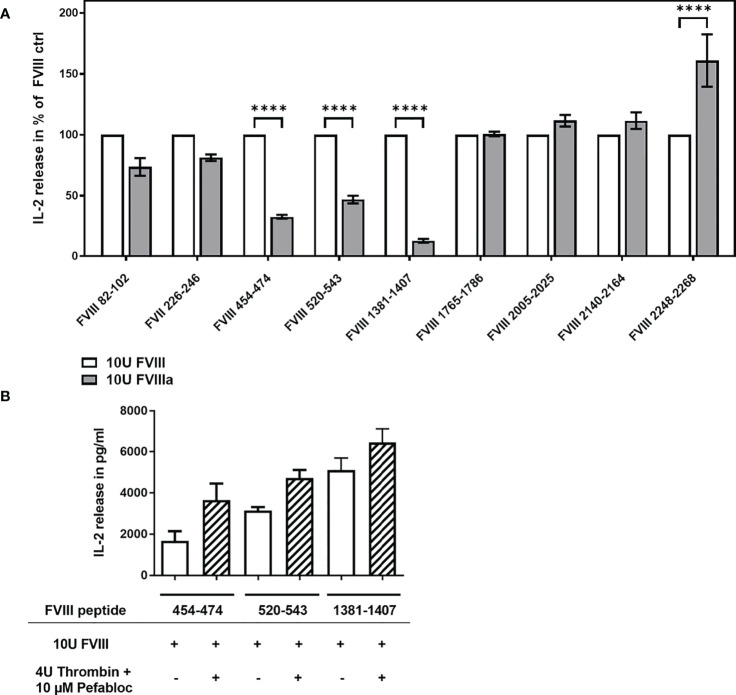
Differential activation of FVIII peptide-specific CD4+ T cells hybridoma clones by FVIII and thrombin-activated FVIIIa, present during preincubation of APCs. FVIII specific CD4+ T cell hybridoma clones were cocultured with APCs preincubated with either FVIII or thrombin-activated FVIIIa. **(A)** Relative IL-2 release by FVIII-specific CD4+ T cell hybridoma clones specific for the different FVIII peptide regions tested, comparing non-activated FVIII (100%) and thrombin-activated FVIIIa, present during the preincubation of APCs prior to subsequent co-culture with CD4+ T cell hybridoma clones. Depicted is the mean value ± SEM. Results were derived from 2 independent experiments done in triplicates (n=6). Statistical analysis for significance between different groups of measured mean IL-2 release was assessed by a two-way ANOVA with Sidaks’s multiple comparison test using Graphpad Prism 9 (GraphPad Software, Inc., California USA). P < 0.05 was considered statistically significant. *p<0.05; **p<0.01; ***p<0.001; ****p<0.0001; **(B)** Control experiment testing a potential influence of Thrombin and Pefabloc, both used for the generation of activated FVIIIa, on APC function. APCs were preincubated with Thrombin and Pefabloc, and afterwards washed before adding FVIII. Subsequently, preincubated APCs were co-cultured with FVIII-specific CD4+ T cell hybridoma clones specific for the FVIII peptides indicated.

The cofactor activity of FVIII during the propagation phase of coagulation is required for the generation of a thrombin burst. FVIII gets activated at the site of bleeding by its physiological activator thrombin leading to the structurally distinct FVIIIa. We were interested to study if thrombin-activated FVIIIa is differently processed and presented by APCs when compared to non-activated FVIII. In the experiments presented in **Figure 4A**, we compared the activation of FVIII-specific CD4+ T cell hybridoma clones by APCs preincubated with either 10U/mL FVIII or 10U/mL FVIIIa. FVIIIa was generated prior the preincubation culture of APCs by the addition of Thrombin which was inactivated after 30min by adding Pefabloc to the culture mixture. The APCs were cultured in the culture mixture for 3 hours to allow for FVIIIa uptake and subsequently washed prior to the co-culture with FVIII-specific CD4+ T-cell hybridoma clones. We observed a substantially reduced activation of FVIII-specific CD4+ T cell hybridoma clones which are specific for peptides in the A2 and B domains (**Figure 4A**). A control experiment which only included Thrombin and Pefabloc in the culture mixture of APCs before adding FVIII indicated that the reduced activation of the respective FVIII-specific CD4+ T cell hybridoma clones were not due to a direct effect of Thrombin or Pefabloc on the activity of APCs (**Figure 4B**).

### Blocking specific receptors on APCs modifies the FVIII peptide repertoire presented on MHC class II, analyzed by the subsequent activation of FVIII-specific CD4+ T cell hybridoma clones

**Figure 5:**
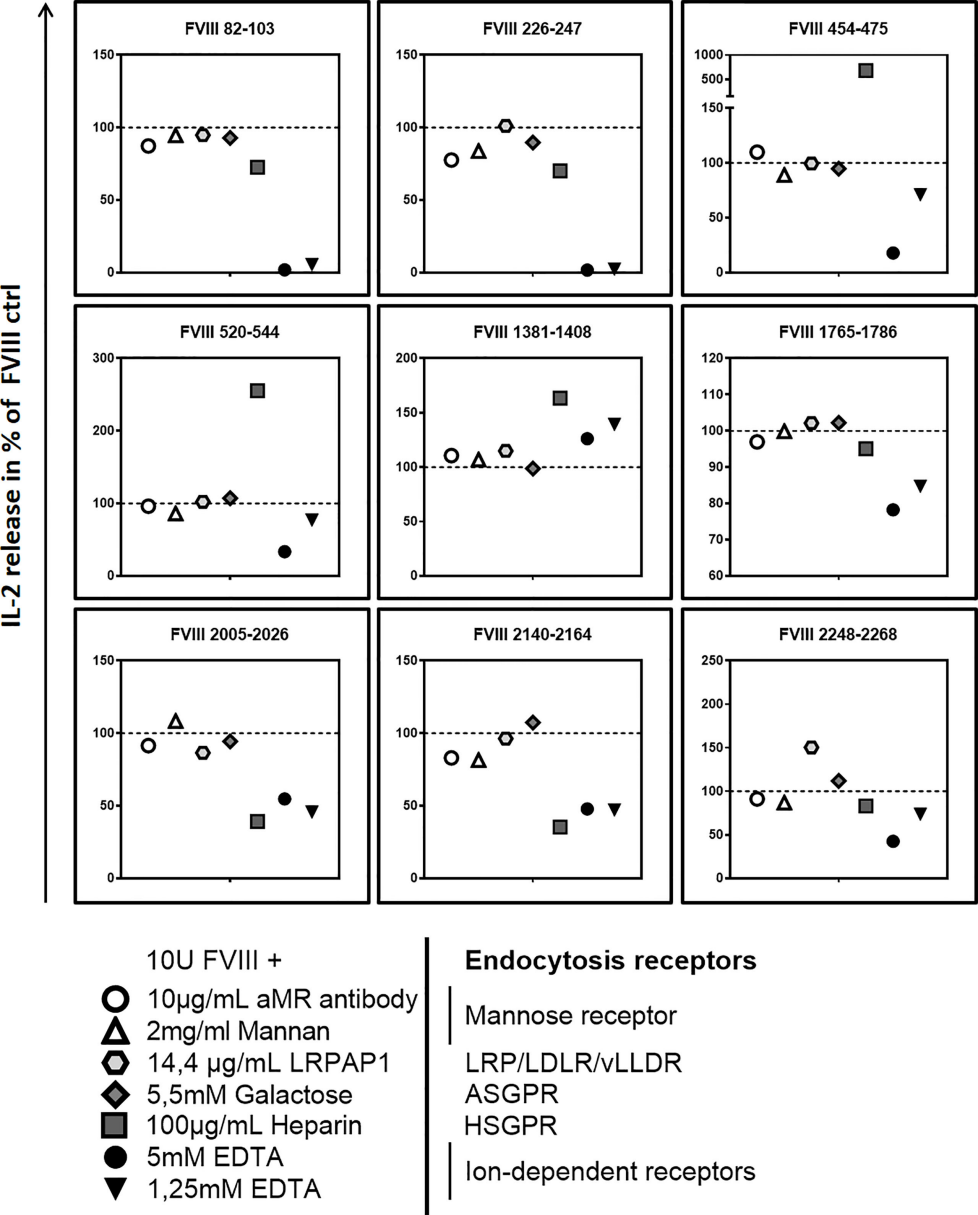
Blocking endocytosis receptors during preincubation of APCs modulates presentation of FVIII-derived peptides and subsequent stimulation of FVIII peptide-specific CD4+ T cells hybridoma clones. APCs were preincubated with specific blocking agents for endocytosis receptors which are associated with the endocytosis of FVIII: 5mM Galactose to block the asialoglycoprotein receptors (ASGPR); 100µg/mL Heparin to block heparan sulfate proteoglycans (HSGP), 2mg/mL Mannan or 10µg/mL of a specific monoclonal antibody to block the Mannose Receptor, 14.4 µg/mL recombinant mouse LRPAP1 to block the low-density lipoprotein receptor–related protein-1 (LRP1), 1.25mM and 5mM EDTA to inhibit the function of bivalent ion-dependent receptors. After 3hrs incubation, APCs were washed and subsequently cocultured with FVIII specific CD4+ T cell hybridoma clones covering the 9 CD4 T-cell epitopes recognized by the hybridoma library. Relative IL-2 release as an indicator for the activation of hybridoma clones in relation to the normal FVIII control (100%) is presented. Depicted is the mean value derived from 2 independent experiments done in triplicates (n=6). Detailed data and statistical analysis are depicted in **Table 3**.

Finally, we asked if blocking specific receptors on APCs which are associated with the uptake of FVIII results in an alteration of the FVIII peptide pattern presented by MHC-class II. Multiple receptors associated with FVIII uptake were previously reported, including mannose receptor (MR), asialoglycoprotein receptors (ASGPR), low-density lipoprotein receptor–related protein-1 (LRP1) and heparan sulfate proteoglycans (HSPG) (16, 25–31). We preincubated APCs with different blocking agents prior to the addition of FVIII. Our results indicate that blocking the MR or the ASGPR by galactose, mannan or a blocking monoclonal anti-MR antibody did not influence the subsequent activation of any of the FVIII peptide-specific CD4+ T cell hybridoma clones (**Figure 5** and **Table 3**). However, when we complemented the culture medium used for preincubation of APCs with recombinant mouse low density lipoprotein receptor-related protein-associated protein 1 (LRPAP1), a specific ligand for LRP1 and other receptor-associated protein (RAP)-sensitive receptors, we observed a significant increase in the subsequent activation of FVIII-specific CD4+ T cell hybridoma clones which are specific for an epitope in the in the C2 domain (FVIII 2248-2268). This increase was reflected by an about 50% higher IL-2 release when compared to controls (**Figure 5** and **Table 3**). However, blocking LRP1 on APCs did not modulate the subsequent activation of any of the CD4+ T cell hybridoma clones specific for the other FVIII peptides investigated, e.g. FVIII 82-102, FVIII 226-246, FVIII 454-474, FVIII 520-543, FVIII 1381-1407, FVIII 1765-1786, FVIII 2005-2025 and FVIII 2140-2164 (**Figure 5** and **Table 3**).

**Table 3:** Influence of different blocking agents for endocytosis receptors present during preincubation of APCs with FVIII, on the peptide specificity of subsequently activated FVIII-specific CD4+ T cell hybridoma clones .

FVIII T-cell epitope	10U FVIII +							
	aMR antibody (10µg/mL)	Mannan (2mg/mL)	LRPAP1 (14.4 µg/mL)	Galactose (5.5mM)	EDTA (5mM)	EDTA (1.25mM)	Heparin(100µg/mL)
	Il-2 release in % of FVIII ctrl ( ± SEM)							
FVIII 82-102	87.33 ( ± 2.55)	94.61 ( ± 3.15)	94.81 ( ± 7.40)	92.87 ( ± 4.20)	1.92 ( ± 0.19)****	5.49 ( ± 0.39)****	72.67 ( ± 2.40)
FVII 226-246	77.61 ( ± 1.36)	83.85 ( ± 5.02)	101.11 ( ± 6.82)	89.71 ( ± 3.69)	1.77 ( ± 0.18)****	2.39 ( ± 0.33)****	70.09 ( ± 7.41)
FVIII 454-474	109.81 ( ± 8.20)	88.94 ( ± 9.42)	99.38 ( ± 4.58)	94.76 ( ± 5.85)	17.98 ( ± 1.97)****	71.03 ( ± 7.52)	680.76 ( ± 86.09)****
FVIII 520-543	96.36 ( ± 7.71)	86.11 ( ± 3.38)	102.24 ( ± 4.73)	107.14 ( ± 5.61)	33.46 ( ± 2.48)***	76.99 ( ± 4.28)	255.32 ( ± 6.67)****
FVIII 1381-1407	110.70 ( ± 3.87)	106.93 ( ± 7.52)	114.94 ( ± 4.96)	98.61 ( ± 4.71)	126.16 ( ± 3.79)	139.20 ( ± 1.79)	163.30 ( ± 8.88)**
FVIII 1765-1786	96.95 ( ± 2.12)	99.86 ( ± 3.00)	102.14 ( ± 2.11)	102.24 ( ± 1.79)	78.24 ( ± 2.44)	84.75 ( ± 3.70)	95.09 ( ± 2.07)
FVIII 2005-2025	91.54 ( ± 5.66)	108.43 ( ± 3.38)	86.52 ( ± 5.16)	94.38 ( ± 4.21)	54.76 ( ± 6.69)	45.69 ( ± 6.76)*	39.28 ( ± 2.74)**
FVIII 2140-2164	83.11 ( ± 7.14)	81.57 ( ± 8.12)	96.32 ( ± 7.74)	107.43 ( ± 9.14)	47.90 ( ± 5.90)*	47.12 ( ± 7.21)*	35.37 ( ± 1.90)**
FVIII 2248-2268	91.26 ( ± 11.89)	86.84 ( ± 6.56)	150.34 ( ± 18.41)*	111.97 ( ± 25.64)	42.60 ( ± 7.82)**	73.69 ( ± 15.59)	83.09 ( ± 11.33)

aMR antibody and mannan: to block mannose receptors (MR); LRPAP1 to block LDL receptor family members and LDL receptor-related protein (LRP); Galactose to block asialoglycoprotein receptors (ASGPR); EDTA to inactivated ion-dependent receptors; Heparin to block heparan sulfate proteoglycans (HSPG); FVIII ctrl: FVIII only control group. Presented is the relative IL-2 release, compared to the IL-2 release in the respective “FVIII only” control group (100%). Statistical analysis for significance between different groups of measured mean IL-2 release was assessed by a two-way ANOVA with Sidaks’s multiple comparison test using Graphpad Prism 9. P < 0.05 was considered statistically significant. *p<0.05; **p<0.01; ***p<0.001; ****p<0.0001.

Pretreatment of APCs with heparin to block binding of FVIII to HSPG reduced the subsequent activation of FVIII-specific CD4+ T cell hybridoma clones which are specific for peptides in the A3 and C1 domains of FVIII (FVIII 2005-2025 and FVIII 2140-2164) but substantially increased the subsequent activation of FVIII-specific CD4+ T cell hybridoma clones which are specific for peptides in the B and A2 domains of FVIII (FVIII 1381-1407, FVIII 520-554 and FVIII 454-474) (**Figure 5** and **Table 3**). Pretreatment of APCs with EDTA, which functionally blocks bivalent ion-dependent receptors, resulted in the substantial reduction of the subsequent activation of FVIII-specific CD4+ T cell hybridoma clones which are specific for peptides in the A1 and A2 domains of FVIII (FVIII 82-102, FVIII 226-246, FVIII 454-474 and FVIII 520-543) and a mild to moderate reduction in the activation of hybridoma clones specific for peptides in the C1 and C2 domains (FVIII 2005-2025, FVIII 2140-2164 and FVIII 2248-2268) (**Figure 5** and **Table 3**). In contrast, preincubation of APCs with EDTA did not significantly alter the subsequent activation of hybridoma clones which are specific for peptides in the A3 domain (FVIII 1765-1786) or for peptides in the B domain (FVIII 1381-1407).

## Discussion

This *in vitro* study assessed the FVIII peptide specificity of an activated FVIII-specific HLA-DRB1*1501 restricted CD4+ T-cell hybridoma library in relation to different microenvironment conditions during FVIII endocytosis by APCs. The results of this study indicate that modifications of the microenvironment during FVIII uptake by APCs alter the peptide specificity of subsequently activated FVIII-specific CD4+ T cell hybridoma clones. Based on these data we hypothesize that the route of FVIII uptake by APCs and the subsequent routing into different endosomal compartments might determine the FVIII peptide repertoire presented on MHC-class II expressed by APCs and the peptide specificity of subsequently activated FVIII-specific CD4+ T cells.

We used a FVIII-specific CD4+ T cell hybridoma library derived from humanized E17 HLA-DRB1*1501 mice as a sensitive *in vitro* tool to study alterations in the FVIII peptide specificity of activated FVIII-specific CD4+ T cells in relation to alterations in the microenvironment during FVIII uptake by APCs. FVIII is most likely taken up by a range of different APCs *in vivo*. Therefore, we decided to use total splenocytes, containing different APC populations present *in vivo*, as APCs for the major part of our studies. Our aim was to obtain a general understanding if and how the microenvironment of APCs during FVIII uptake influences the subsequently generated FVIII peptide repertoire. However, as different APC populations might uptake and process FVIII differently, we were interested if major APC populations present in mouse splenocytes are capable to activate the FVIII-specific CD4+ T cell hybridoma library. Data obtained with purified splenic CD11c high MHC class II+ dendritic cells, splenic CD11b+ monocytes/macrophages and splenic CD19+ B cells show that only quantitative, but not qualitative changes in the Il-2 response of CD4+ T cell hybridoma clones, indicating that the different APC populations present in whole splenocytes are able to present all identified FVIII peptides to the FVIII-specific CD4+ T cell hybridoma library. Interestingly, although splenic CD11c high MHC class II+ dendritic cells expectedly outperformed splenic CD11b+ monocytes/macrophages and splenic CD19+ B cells in the capacity to activate most of the FVIII-specific CD4+ T cell hybridoma clones tested, all purified APC populations were similarly able to activate clones recognizing FVIII peptide 1765-1786 in the A3 domain.

We would like to emphasize several limitations associated with the utilization of the hybridoma library. The FVIII peptides presented on APCs in the context of MHC-class II are restricted to the human HLA-DRB1*1501 haplotype. Furthermore, using this library, only 9 different FVIII T-cell epitopes could be identified. Consequently, the FVIII-specific CD4+ T cell hybridoma library can only cover a proportion of the FVIII T-cell epitopes found in the total population of patients with severe hemophilia A. However, the particular haplotype HLA-DRB1*1501 was reported to be associated with an increased risk for patients with severe hemophilia A to develop FVIII inhibitors following FVIII replacement therapies (40–42). Moreover, van Haren and colleagues demonstrated, that 6 peptide specificities identified in our FVIII-specific CD4+ T cell hybridoma library overlap with peptides presented on human APCs after pulsing with FVIII (43). Therefore, we believe that the FVIII-specific CD4 T-cell epitopes investigated in our studies are relevant for patients. Another limitation of our model is the fact that it includes murine APCs expressing the human MHC-class II. We cannot exclude that the murine nature of the APCs is associated with inherent differences in processing and presentation of antigenic peptides when compared to their human APC counterparts. Previously we compared APCs derived from the humanized HLA-DRB1*1501 mouse model with human monocyte-derived dendritic cells generated from a healthy blood donor who was homozygous for the MHC-class II haplotype HLA-DRB1*1501. We could show that both types of APCs were equally able to stimulate the FVIII-specific CD4+ T cell hybridoma library derived from the humanized HLA-DRB1*1501 mouse model (32). Therefore, we believe that our study can serve as a proof of principle demonstrating that the microenvironment during FVIII uptake by APCs shapes the repertoire of FVIII peptides presented to CD4+ T cells.

After secretion, FVIII immediately forms a non-covalent complex with VWF, whose potential role on the immunogenicity of FVIII has been under intense discussion over the last decade (44). Previous studies proposed VWF to affect FVIII immunogenicity by significantly reducing FVIII endocytosis (14–16). The data presented in our study suggests that VWF does not inhibit the general uptake of FVIII by APCs, but has distinct effects on the presentation of some FVIII peptides but not on others. These findings are in line with data previously reported by Sorvillo et al. indicating that VWF modulates the internalization and presentation of FVIII in immature human dendritic cells, using mass spectrometry analysis of the eluted MHC class II – peptide complex (15).

Our data demonstrate that cleavage and activation of FVIII by thrombin leads to an altered FVIII peptide repertoire presented on APCs. This is particularly interesting as it indicates that activated FVIII as generated at bleeding sites is processed differently than non-activated FVIII. We believe that FVIIIa binds to different endocytosis receptors expressed on APCs and, therefore, might be routed to different endosomal compartments when compared to non-activated FVIII. These differences could result in modifications of the FVIII peptide repertoire presented by APCs. In line with this hypothesis, Bovenschen et al. previously showed that proteolytic cleavage of factor VIII is required to expose the binding-site for low-density lipoprotein receptor related protein within the A2 domain of FVIII (45).

To further investigate the association between different microenvironment conditions during FVIII uptake and the FVIII peptide patterns presented by APCs, we blocked surface receptors on APCs which are believed to be associated with the uptake of FVIII. Several receptors have been identified to play an important role in the clearance of FVIII, such as mannose receptor (MR), asialoglycoprotein receptors (ASGPR), low-density lipoprotein receptor–related protein-1 (LRP1) and heparan sulfate proteoglycans (HSPG) (16, 25–31). However, it is not completely understood if these receptors are also involved in FVIII uptake by APCs. Based on the data of our study, we conclude that neither blocking the asialoglycoprotein receptors nor blocking the mannose receptor seem to alter the FVIII peptide repertoire presented by APCs which is in line with previous findings by Dasgupta and Herczenik (16, 26). Although LDL receptor family members and LDL receptor-related proteins (LRP) are important receptors for FVIII clearance (46, 47), they have been reported not to influence the capacity of human APCs for FVIII uptake (16, 26). Data of our study indicate that blocking the LRP on APCs modifies the subsequent activation of a CD4+ T cell hybridoma clone that is specific for a peptide in the C2 domain of FVIII. When blocking bivalent ion-dependent receptors expressed on APCs with EDTA, the subsequent activation of most of the CD4+ T cell hybridoma clones was down-regulated with the exception of clones recognizing peptides in the A3 domain (FVIII 1765-1786) or peptides in the B domain (FVIII 1381-1407), which were not significantly altered in their magnitude of activation. This data suggest that FVIII endocytosis by APCs does not seem to be completely blocked by EDTA as previously suggested (14). Moreover, FVIII uptake by APCs seem to involve both ion-dependent and ion-independent mechanisms. HSPG has been shown to effectively bind and concentrate FVIII on the cell surface of APCs and thereby facilitates endocytosis of FVIII by endocytosis receptors such as e.g. LRP (31). Competing with HSPG binding using heparin resulted in pronounced changes in the magnitude of activation of the FVIII-specific CD4+ T cell hybridoma clones. The question remains if these changes are due to a reduction of FVIII endocytosis by APCs or to an uptake of FVIII *via* different endocytosis receptors, or to a mixture of both mechanisms.

The data of our study suggest a crucial relationship between the specific microenvironment during FVIII endocytosis by APCs and the FVIII peptide repertoire presented by APCs. Using BS-C-1 cells, it was previously demonstrated, that endocytosed proteins are not generally sorted into a common pool of early endosomes, but rather into different subsets of endosomes (48) which differ in their specialized functions (49–53). Moreover, it was shown that the sorting of endocytosed proteins into different compartments of the endosomal pathway is dependent on the endocytosis receptor (54). Different endosomal compartments do not only contain different sets of enzymes for protein processing but also different quantities of available class II molecules for peptide loading (13). Based on those findings and based on the results of our own study, we hypothesize that the diversity of the FVIII peptide repertoire presented by APCs to CD4+ FVIII-specific T cells does not only depend on the type of APC but also on the local microenvironment at the time of FVIII uptake by APCs. In this context, uptake of FVIII and FVIIIa by APCs at local bleeding sites might result in a different peptide repertoire presented to FVIII-specific CD4+ T cells when compared to the uptake of FVIII by APCs at non-bleeding sites. It is well established that some patients with hemophilia A repeatedly bleed into their joints, creating a chronic inflammatory microenvironment (55). It can be expected that such environment will not only facilitate the recruitment of more APCs to the site of inflammation but will also influence antigen processing itself as described for human dendritic cells (56). Fiebiger et al. presented data indicating that the balance of pro- and anti-inflammatory cytokines can directly affect the antigen presentation capacity of APCs by modifying the biosynthesis, stability or activity of the enzymes involved in endocytic proteolysis (56).

In conclusion, our data suggest that the the microenvironment during FVIII endocytosis by APCs determines the FVIII peptide repertoire presented on MHC-class II and, therefore, the specificity of subsequently activated FVIII-specific CD4+ T cells. Whether alterations in the FVIII peptide repertoire presented on APCs results in subsequent alterations in the induction of neutralizing or non-neutralizing antibodies against FVIII requires further studies.

## Data availability statement

The raw data supporting the conclusions of this article will be made available by the authors, without undue reservation.

## Ethics statement

The animal study was reviewed and approved by Institutional Animal Care and Use Committee of Baxalta Innovations GmbH, a member of the Takeda group of companies, Vienna, Austria.

## Author contributions

Contribution: CL designed research, performed *in vitro* analysis, analyzed and interpreted data, and wrote the manuscript. KS and PH designed research, and analyzed and interpreted data. BH and TP performed *in vitro* analysis and interpreted data. MW designed, supervised, and performed animal experiments. BR designed research, analyzed and interpreted data, and wrote the manuscript.

## Acknowledgments

The authors thank Elisabeth Hopfner for technical assistance and Maria Schuster for supervising animal breeding. The authors also thank Fritz Scheiflinger who supported part of the study.

## Conflict of interest

MW is an employee of Baxalta Innovations GmbH, a member of the Takeda group of companies, Vienna, Austria.

CL, KS, BH, TP, PH, and BR were employees of Baxalta Innovations GmbH, a member of the Takeda group of companies, Vienna, Austria, at the time when this study was done.

The authors declare that this study received funding from Baxalta Innovations GmbH. The funder had the following involvement in the study: study design, interpretation of data, writing of this article and the decision to submit it for publication.

## Publisher’s note

All claims expressed in this article are solely those of the authors and do not necessarily represent those of their affiliated organizations, or those of the publisher, the editors and the reviewers. Any product that may be evaluated in this article, or claim that may be made by its manufacturer, is not guaranteed or endorsed by the publisher.
